# Factors associated with aberrant imprint methylation and oligozoospermia

**DOI:** 10.1038/srep42336

**Published:** 2017-02-10

**Authors:** Norio Kobayashi, Naoko Miyauchi, Nozomi Tatsuta, Akane Kitamura, Hiroaki Okae, Hitoshi Hiura, Akiko Sato, Takafumi Utsunomiya, Nobuo Yaegashi, Kunihiko Nakai, Takahiro Arima

**Affiliations:** 1 Department of Informative Genetics, Tohoku University Graduate School of Medicine, 2-1 Seiryo-cho, Aoba-ku, Sendai, 980-8575, Japan; 2 Laboratory of Animal Reproduction and Development, Graduate School of Agricultural Science, Tohoku University, 1-1 Amamiya-machi, Tsutsumidori, Aoba-ku, Sendai, 981-8555, Japan; 3 Department of Development and Environmental Medicine, Tohoku University Graduate School of Medicine, Sendai, 980-8575, Japan; 4 St. Luke Clinic, Oita, 870-0823, Japan; 5 Departments of Obstetrics and Gynecology, Tohoku University Graduate School of Medicine, Sendai, 980-8574, Japan

## Abstract

Disturbingly, the number of patients with oligozoospermia (low sperm count) has been gradually increasing in industrialized countries. Epigenetic alterations are believed to be involved in this condition. Recent studies have clarified that intrinsic and extrinsic factors can induce epigenetic transgenerational phenotypes through apparent reprogramming of the male germ line. Here we examined DNA methylation levels of 22 human imprinted loci in a total of 221 purified sperm samples from infertile couples and found methylation alterations in 24.8% of the patients. Structural equation model suggested that the cause of imprint methylation errors in sperm might have been environmental factors. More specifically, aberrant methylation and a particular lifestyle (current smoking, excess consumption of carbonated drinks) were associated with severe oligozoospermia, while aging probably affected this pathology indirectly through the accumulation of PCB in the patients. Next we examined the pregnancy outcomes for patients when the sperm had abnormal imprint methylation. The live-birth rate decreased and the miscarriage rate increased with the methylation errors. Our research will be useful for the prevention of methylation errors in sperm from infertile men, and sperm with normal imprint methylation might increase the safety of assisted reproduction technology (ART) by reducing methylation-induced diseases of children conceived via ART.

Male infertility is the cause of approximately half of human infertility and recently the incidence has increased in industrialized countries, including Japan (Japan Society of Obstetrics and Gynecology Registry). Oligozoospermia, which is characterized by low concentrations of spermatozoa in the semen, is the most common form of male infertility. Although different genetic alterations are known in oligozoospermia, the number thus far identified is small^1^,^2^,^3^. It has been suggested that defects in spermatogenesis are associated with epigenetic abnormalities in DNA methylation and histone modifications^4^,^5^ We, and others, have shown that abnormal spermatogenesis is associated with aberrant DNA methylation of the imprinted germline differentially methylated regions (gDMRs), particularly in sperm samples from men with oligozoospermia^6^,^7^,^8^,^9^,^10^.

Genomic imprinting is an epigenetic phenomenon that describes parent-of-origin patterns of monoallelic gene expression^11^. This monoallelic expression is established by DNA methylation of CpG-dinucleotides in one or the other germline which then form gDMRs after fertilization. These gDMRs act as imprint control regions (ICRs) that generate extensive domains of imprinted chromatin, some of which span several megabases. The DNA methylation of imprinted gDMRs is reset with every reproductive cycle during gametogenesis^12^,^13^. Ejaculated and mature sperm should be methylated in the paternal gDMRs (pgDMRs), but unmethylated in the maternal gDMRs (mgDMRs). Over 100 genes in humans are regulated by genomic imprinting, and many of these have critically important roles in early development^11^. The failure to correctly establish or maintain imprints causes rare but striking childhood development imprinting disorders and, later in life, both metabolic and behavioral diseases^14^,^15^,^16^.

Epigenetic modifications are influenced by external environmental factors such as endocrine disruptors (including persistant organic pollutants, POPs), certain foods, and drug exposures particularly during embryogenesis and gametogenesis^4^,^5^,^17^. Several POPs, e.g. PCBs, that persist in the environment have been shown to have toxic effects on reproductive and endocrine functions in humans^18^. A number of human epidemiological studies have demonstrated the adverse effects of exposure to POPs on markers of reproduction, including semen quality (sperm concentration, motility and morphology)^19^,^20^,^21^, spermatic DNA integrity^22^,^23^ and circulating reproductive hormone levels^19^.

Epigenetic marks can also be influenced by the diet during fetal development^24^,^25^,^26^. Agouti viable yellow (Avy) is a fascinating animal model whereby the environmental influences on the epigenome can be monitored via a coat color phenotype^27^. The extent of this coat color change is influenced by the degree of methylation of the IAP element, which can be influenced by methyl donor supplementation of the maternal diet^28^. Maternal and post-weaning high-fat diets can also alter epigenetic regulation of the hedonic reward pathways and metabolic regulation of the energy balance in mice^29^, as well as methylation of the leptin promoter in rats^30^. These data provide compelling evidence that diet alone can alter the epigenome^31^. However, we do not know why imprint methylation errors are present in sperm. It is possible that environmental factors and lifestyle exposures to nutritional and reproductive toxicants during gametogenesis contribute to both the sperm properties and epigenetic defects. Given that the use of ART protocols employing sperm from subfertile men is on the increase, understanding the origins of defects in sperm is of paramount importance.

In this study, we aimed to clarify the influences of environmental factors on imprint methylation in human sperm. We identified some lifestyles (lack of exercise and not living in a farming or fishing village) that were associated with aberrant imprint methylation. This aberrant imprint methylation, the identified lifestyles and known factors (aging, current smoking, consumption of carbonated drinks and accumulation of PCBs) could cause a decrease of the sperm concentration. In addition, we examined the relationship between methylation errors in the sperm and pregnancy outcomes.

## Results

### Analysis of imprinted methylation in sperm from normal and oligozoospermic men

Background data of male subjects are presented in Table 1. A total of 221 male partners in infertile couples took part in our study and were classified according to the sperm concentration: normozoospermia (n = 151), moderate oligozoospermia (n = 40) and severe oligozoospermia (n = 30). Although the patients with severe oligozoospermia had the highest levels of both luteinizing hormone (LH) and follicle-stimulating hormone (FSH), the mean ages and physical status (e.g., height, weight and BMI) were similar among the three groups (Table 1).

We analyzed the primary DNA methylation patterns of 3 paternally and 19 maternally methylated gDMRs in 221 sperm DNA samples using the COBRA method. We previously determined that the cutoff value for aberrant sperm DNA methylation of this method was less than 80% of pgDMRs and more than 20% of mgDMRs^9^, therefore we defined the cutoff values of 80% and 20%, respectively, as detection limits of this COBRA method. We identified abnormal methylation samples at one or more loci. We found abnormal methylation in 55 patients (24.8%) (Table 1, Supplemental Figure S1). In our sperm analysis, we found aberrant methylation levels in 25 of the 151 patients (16.6%) with normozoospermia, 9 of the 40 patients (22.5%) with moderate oligozoospermia and 21 of the 30 patients (70.0%) with severe oligozoospermia (Table 1) and aberrant methylation was associated with oligozoospermia (*P* < 0.05, chi-squared test), similar to the findings of our two previous independent studies^8^,^9^. These results confirmed the association between oligozoospermia and defective imprint methylation. There was a tendency for imprint methylation errors to be present at a higher frequency in sperm samples with lower motility and high rates of malformation (Table 1, Supplemental Figure S1). These striking associations of aberrant DNA methylation and sperm properties might suggest that a normal DNA methylation pattern is important for ontogeny.

### Factors related to aberrant imprint methylation in sperm

DNA methylation might be influenced by physical parameters such as age, BMI and/or by environmental factors such as endocrine disruptors, certain foods, drink and drug exposures during gametogenesis. Using information from questionnaires, we examined the associations between physical parameters and environmental factors (e.g., lifestyle and food intake) (Supplemental Table S3) and the presence of imprint methylation errors (Supplemental Table S1). The mean ages (±standard deviations; SD) of patients without and with abnormal sperm methylation were 35.4 (±5.4) years and 36.5 (±6.1) years, respectively. Of the 46 physical and environmental factors investigated, two environmental factors showed a significant difference between normal and abnormal sperm methylation. The rate of those who did not exercise habitually was higher than for those with normal sperm methylation (*P* < 0.05, chi-squared test) (Supplemental Table S1). We also found that the place of residence (not living in a farming or fishing village) was associated with abnormal sperm methylation (*P* < 0.05, chi-squared test) (Supplemental Table S1).

Next, we measured the PCB concentrations in 113 randomly selected serum samples taken from men who participated in this study on the same day as the sperm analysis was performed. The average PCB concentration was 61.1 ng/g lipids, with a range from 12.2 to 247.1 ng/g lipids. We then compared the associations between the PCB concentration and the presence of imprint methylation errors (Supplemental Table S1). However, there was no substantial difference in the total PCBs and some of its isoform concentrations between normal and abnormal methylation samples.

### Factors related to decreased sperm concentrations

Of the 70 oligozoospermia patients, 30 had imprint methylation errors. Therefore we examined factors that may be associated with abnormal sperm concentrations (Table 2, Supplemental Table S2). Although no factor associated with abnormal sperm concentrations was determined from the answers to the questionnaires, we found that serum PCB concentrations were statistically significant (*P* < 0.05, chi-squared test and Kruskal-Wallis test). In addition, there were significant correlations of most PCB isoform concentrations with sperm concentrations (Table 2).

### Multivariable analysis of severe oligozoospermia, aberrant methylation and their related factors

**Figure 1:**
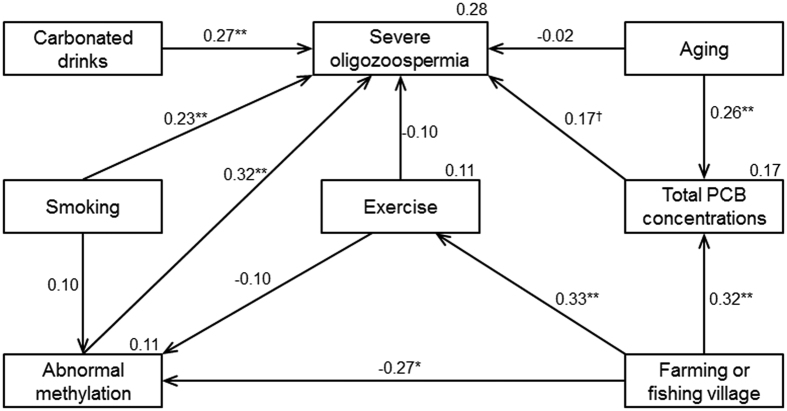
Multivariable analysis of severe oligozoospermia, aberrant methylation and related factors using structural equation modeling. Model results in good overall fit (χ^2^(16) = 10.546 (p = 0.837), GFI = 0.971, AGFI = 0.934, CFI = 1.000, RMSEA = 0.0001). Standardized coefficients are shown above the arrows. Coefficients of determination are shown at the upper right of each factor (severe oligozoospermia abberant methylation, total PCB concentrations and habitual exercise). ^†^*P* < 0.10, *P < 0.05, **P < 0.01.

The environmental factors, residence, exercise and PCB concentrations, related to the aberrant methylation and the decreased sperm concentrations were identified by univariate analysis (shown in Table 2 and Supplemental Table 1). Previous studies reported that several critical factors (i.e., aging^5^,^31^,^32^, smoking^33^ and carbonated drinks^34^,^35^) were associated with sperm concentrations. We next examined the relationship between the most decreased sperm concentrations (severe oligozoospermia), aberrant methylation and environmental factors. Drawing on the hypothesized model and significant correlations from the multivariable analysis, the following variables were used to construct a model: severe oligozoospermia, abnormal methylation, aging, total PCB concentrations, farming or fishing village, exercise, smoking and carbonated drinks (Fig. 1). The fit indices of this model were excellent: chi-square χ^2^(16) = 10.546 (p = 0.837), GFI = 0.971, AGFI = 0.934, CFI = 1.000, RMSEA = 0.0001. This model suggested that aberrant methylation had an inverse correlation with the place of residence (living in a farming or fishing village) (β_std_ = −0.27; *P* = 0.021). Severe oligozoospermia was related to drinking carbonated drinks more than 3 times per week (β_std_ = 0.27; *P* = 0.003), currently smoking (β_std_ = 0.23; *P* = 0.014), aberrant methylation (β_std_ = 0.32; *P* < 0.001) and an increased total PCB concentrations (β_std_ = 0.17; *P* = 0.070). Aging and the place of residence were related to increased total PCB concentrations (β_std_ = 0.26; *P* = 0.007 and β_std_ = 0.32; *P* = 0.001, respectively), and could be indirectly associated with severe oligozoospermia. However, there was no significant association between aberrant methylation/severe oligozoospermia and the habit of exercise.

### Pregnancy outcomes of ART-treated patients with imprint methylation errors and low concentrations of sperm

Some of the subjects who participated in this study were undergoing ART treatment (IVF and/or ICSI), which provided the opportunity to examine pregnancy outcomes from the sperm samples we had characterized. A total of 219 cases of ART treatment resulted in 33 miscarriages, 43 live births and 76 pregnancies (Supplemental Table S3). Live-birth rates (live births/ART treatments) were lower and miscarriage rates (miscarriages/ART treatments) were higher when methylation errors were present in sperm (Table 3, Supplemental Table S3). This might suggest that these methylation errors in sperm contributed to poor pregnancy outcomes. According to the sperm concentration, the lowest live-birth rate and the highest miscarriage rate were also associated with patients who had severe oligozoospermia (Table 3).

## Discussion

There have been many reports about the cause of decreased sperm numbers in men^36^,^37^, but there are few publications demonstrating an association with epigenetic alterations in sperm^38^,^39^. In this study, we examined what lifestyle factors, including serum PCB concentrations, may lead to aberrant DNA methylation in human sperm. We identified some lifestyles (lack of exercise and not living in a farming or fishing village) that could contribute to the aberrant imprint methylation. In addition, based on the structural equation model, this aberrant imprint methylation, the identified lifestyles, and known factors (aging, current smoking, consumption of carbonated drinks and accumulation of PCBs) could lead to decreased sperm concentrations.

### Characterization of aberrant imprint methylation in sperm

We found imprint methylation alterations in 24.8% of the sperm samples from 221 patients. The most frequent methylation errors were at the *GTL2*-DMR (10.6%) and *PEG3*-DMR (9.1%), similar to our previous findings^8^,^9^. Although the most frequent methylation errors were shown to be at the *PEG1*-DMR in our previous papers, the frequency was lower in this study. This may suggest that these regions are simply stochastic or that these imprinted regions are prone to methylation errors.

Eight individuals had more than 10 genes that had aberrant methylation levels compared to the majority of samples, which had 1–3 methylation errors. Six of these samples exhibited severe oligozoospermia and 2 normozoospermia. We speculated that there might be a different etiology, independent of the sperm concentration, in patients with a high number of methylation errors. However, the reason for the high number of methylation errors could not be determined.

### Factors contributing to imprinted methylation errors in sperm

First, a lack of regular exercise and not living in a farming or fishing village have been shown to alter the sperm epigenome. Generally, lack of exercise has an adverse effect on physical health^40^,^41^. Through continuous and moderate exercise, some diseases such as obesity, diabetes and hypertension can be prevented. As with exercise, living in a rural area is expected to lead to physical health, because we found that there was strong correlation between the habit of exercise and living in such areas. A recent study revealed that DNA methylation changes occurred after 3 months of exercise training^42^. Though the functional implications of the exercise-induced changes in pgDMR and mgDMR methylation are unclear, continual and proper exercise might improve sperm DNA methylation patterns. Second, some organic pollutants, used intensively worldwide for several decades until the 1980s, are stable and bioaccumulate in the environment, exposing human populations through their consumption in food. Several POPs have been shown to have toxic effects on reproductive and endocrine functions in humans^18^,^43^. Some studies have indicated adverse effects of PCB exposure on markers of male reproduction, including semen quality (sperm concentration, motility and morphology)^19^,^20^,^21^, sperm DNA integrity^22^,^23^ and circulating reproductive hormone levels^19^ while others reported marginal effects or none^44^,^45^. A recent study of serum PCB concentrations and IVF outcomes suggested an increase in implantation failure with higher levels of serum PCB isoforms^46^. In this study, we found that PCB concentrations were associated with the effects directly on sperm quantity but not epigenetic quality. This may have been due to the limited sample size. Third, carbonated drinks are viewed by many as a major contributor to health problems^47^. It is known that the sugar and caffeine in carbonated drinks worsen the quality of sperm^34^,^35^. Previous studies have revealed that highly sugar-sweetened drinks are associated with a low sperm concentration^34^, and a high cola and/or caffeine intake reduces the sperm concentration and total sperm count^35^. Although we did not investigate the sugar and/or caffeine intakes per day, our result was very similar to these in the studies cited above. Fourth, smoking harms many organs of the body, causes many diseases and reduces the health of smokers in general. According to previous studies, smoking reduces sperm quality (i.e., motility, concentration, vitality and DNA integrity)^33^,^48^. We also confirmed that current smoking strongly affected decreased the sperm concentration, similar to the results of previous studies. In addition, most of the patients with low sperm concentrations had abnormal imprinting methylation. Finally, there may be many other external and internal factors that cause epigenetic alterations. We did not investigate the effects of social stress or drugs in our study.

### Methylation errors in sperm and associated diseases

ART such as ICSI and IVF is widely used for infertility treatments, especially in the case of oligozoospermia. It is already known that there is an association between ART such as ICSI and increased incidences of normally rare imprinting disorders, especially Beckwith-Wiedemann syndrome (OMIM 130650), Angelman syndrome (OMIM 105830) and Silver-Russell syndrome (OMIM 180860)^49^,^50^,^51^. The identification of epigenetic changes at imprinted loci in infants born after ART treatments led to the suggestion that the technique itself may predispose embryos to acquire imprinting errors. However, our work and that of others suggests that epigenetic risks linked to ART techniques can sometimes originate in the use of sperm with preexisting epigenetic errors. This hypothesis was supported by the presence of the same imprinting mutations in failed ART conceptuses and the parental sperm^52^. In this study, we found adverse pregnancy outcomes when there were methylation errors in the sperm. This aberrant methylation in sperm might not only affect fertility and pregnancy, but could lead to various diseases later in life^53^,^54^,^55^,^56^,^57^,^58^. Therefore, methylation analysis of sperm provides useful information and has the potential to substantially reduce the various risks in children conceived via ART.

### Our hypothesis on the epimutation in the sperm

Recent epidemiologic studies have identified advanced paternal age as a risk factor for autism, depression, epilepsy and some cancers in children^59^,^60^. In another recent study, methylation abnormalities of aging sperm were found to be associated with schizophrenia and bipolar disorder^61^. Taken together, these data suggest the possibility that the accumulation of epigenetic errors in the sperm as men age contributes to disease^5^,^31^,^32^. In general, the sperm of aged men are affected by accumulated exposures to environmental and nutritional factors such as reproductive toxicants, certain foods and drug exposures during gametogenesis. However, research on sperm of aged men is just beginning and the mechanism of sperm methylation errors has not yet been clarified. With aging, the sperm count gradually decreases and the rate of oligozoospermia increases. Consequently, sperm with abnormal methylation alterations resemble those of oligozoospermia. Several studies have demonstrated methylation errors of imprinted and non-imprinted genes^62^,^63^. The sperm of aged men and progressive oligozoospermic sperm might have similar epigenetic features so this might be a physiological phenomenon.

In contrast, regardless of aging, our findings suggested that two lifestyles (a lack of regular exercise and not living in a farming or fishing village) could worsen the epigenetic quality of sperm. Although it is unclear what molecular mechanisms adversely affect the sperm methylation levels in the absence of these lifestyles, we inferred that maintaining good physical health might produce the desired therapeutic effect for the epigenetic quality of sperm. In addition, the improvement of this epigenetic quality and modification of lifestyles (moderate or less consumption of carbonated drinks and quitting smoking) might lead to preventive medicine for oligozoospermia. Further studies will be required to verify the relationships among the epigenetic quality of sperm, aging and physical health.

### Limitations of our study

There are some limitations in this study. First, we carefully confirmed the absence of somatic cells in the isolated spermatozoa microscopically. However, it was difficult to completely exclude the possibility of contamination. Second, the COBRA technique analyzes the methylation status at only one or a few CpG per DMR. However we previously reported that methylation status was similar at every CpG site of DMRs^8^. Recently, single cell methylome analysis has been developed^64^,^65^,^66^. To evaluate the situation more precisely, we should examine methylation at the single-cell level. Third, the PCB concentrations were measured in only a small number of the samples (from approximately one hundred patients) because the measurement of serum PCBs is very expensive. We chose the samples randomly. Finally, our questionnaire only asked about lifestyle and eating habits, not mental stress, the amount of alcohol consumption or drug use. In the future we will need to use a more detailed questionnaire.

### Conclusion

In this study, we found the imprint methylation errors in sperm was associated with living environmental factors. Then, aberrant methylation and a particular lifestyle (current smoking, excess consumption of carbonated drinks) were associated with severe oligozoospermia, while aging probably affected this pathology indirectly through the accumulation of PCB in the patients. Where there were methylation errors in the sperm, we found adverse pregnancy outcomes. In women, the rates of miscarriages and congenital abnormalities of newborns gradually increase from around the age of 35. Similarly, our results may demonstrate that there is a suitable period for fathering children in healthy men. We found that some environmental factors affected the density levels and epigenetic quality of sperm. These factors may have synergistic effects with aging. Both biological and environmental factors can contribute to epigenetic alterations in sperm that may contribute to various diseases. It will now be important to determine whether altering particular biological and/or environmental factors can improve the seemingly irreversible decline in the epigenetic quality of sperm.

## Materials and Methods

### Sperm collection

Ejaculated sperm samples were collected from 221 male patients with fertility problems who had consulted a physician at a private clinic. Routine semen analysis for volume, sperm count, rate of motility and morphological abnormalities was performed according to the World Health Organization (WHO) 4^th^ ed. Guideline, 2010. Thereafter, the concentration was calculated from the motile sperm count^67^. Of these patients, 30 had severe oligozoospermia (<5 × 10^6^/mL), 40 had moderate oligozoospermia (5–20 × 10^6^/mL) and the remaining 151 had normal sperm counts (>20 × 10^6^/mL). To prevent somatic cell contamination, we used swim-up methods and purified only sperm. Purification of motile sperm cells and the extraction of the DNA were performed immediately after the routine examination of the ejaculated sperm as described previously^8^. The sperm were washed repeatedly and placed in phosphate-buffered saline and DNA was obtained by using a standard extraction method with the addition of 0.1 mM 2-mercaptoethanol^8^. The remaining fresh sperm samples were washed in a specific medium and kept frozen in liquid nitrogen (−196 °C). The sperm were thawed and used for IVF and/or ICSI.

### Data collection on basic characteristics

We obtained information about the risk factors for imprinting methylation errors in sperm via a questionnaire survey. All questionnaires were filled out at the time of sperm collection. By this method we obtained information on height, body weight, occupation, smoking habits, dietary habits, physical exercise and the reproductive history. There was also a question about the frequency of consumption of 22 food items or beverages.

### DNA methylation analysis

We employed a conventional methylation assay for combined bisulphite PCR restriction analysis (COBRA) of all 22 imprinted gDMRs in sperm DNA. The bisulphite-treated DNA was amplified by PCR and digested with restriction enzymes that cut only if the site was methylated. The primer sequences, restriction enzymes and conditions were described previously^68^.

### Serum PCBs and hormone measurements

Serum samples (5 ml) were collected at the time of sperm examination and stored at −80 °C until analysis. Samples were analyzed for PCB isoforms #70/80, #99, #118, #153, #163/164, #138, #182/187, #180 and #170 by high-resolution gas chromatography/high-resolution mass spectrometry using the isotope dilution method. The laboratory analytical methods and quality control procedures have been described elsewhere^69^. All the analyses were performed by IDEA Consultants, Inc. (Tokyo, Japan). Accuracy was ensured by using a reference serum sample as the quality control; the concentrations of PCBs#138, #153 and #180 were determined to be 0.220, 0.217 and 0.300 μg/L as compared to the reference values (tolerance range) of 0.242 (0.174–0.310), 0.217 (0.152–0.281) and 0.307 (0.222–0.392) μg/L, respectively. The calculated limit of detection (LOD) was 0.03 pg/g-wet, which was identified by the signal-to-noise ratio. When the level of a given PCB congener was below the LOD, half of the LOD value for that congener was imputed. The ∑PCB concentration represented the sum of the all measured congeners, expressed as ng/g-lipid.

Serum concentrations of LH, FSH, testosterone (T) and prolactin (PRL) of the patients were measured using an enzyme immunoassay (EIA) kit.

### Basic characteristics

The basic characteristics of the study subjects are summarized in Table 1. Information on age, height, body weight, body mass index (BMI), sleeping time, the annual income, education level, residence, smoking, alcohol intake and exercise were evaluated for three groups with different sperm concentration properties; normozoospermia, moderate oligozoospermia and severe oligozoospermia. Low family income, defined by the annual revenue of the household, was categorized as an annual income of 4,000,000 Japanese yen or less. A low education level of the couple was defined by the highest academic background and categorized as graduating from high school or less.

### Ethics regarding human subjects

All patients provided written informed consent and the study was approved by the ethical committee of Tohoku University Graduate School of Medicine (Approval number: 2010-120). The study was carried out in accordance with the Ethical Guidelines for Medical and Health Research Involving Human Subjects.

### Statistical analysis

All questionnaires were categorized into two groups, and then differences in the means of these questionnaires were assessed by use of the chi-squared test, Fisher’s exact test or Kruskal-Wallis test. *P* < 0.05 was regarded as significant. Univariate analyses were performed using the JMP Pro 11.0.0 for Windows software package (SAS Institute Inc., Cary, NC).

To investigate the relationship between severe oligozoospermia, aberrant methylation and related factors, the hypothesized model was tested using structural equation modeling conducted with AMOS version 24, with standard maximum likelihood estimation. The fit of the model was examined in terms of the chi-square (CMIN), goodness-of-fit index (GFI), adjusted goodness-of-fit index (AGFI), comparative fit index (CFI), and root mean square error of approximation (RMSEA). The indices of the fit of the model to the data were evaluated with several statistics: GFI of 0.95 or greater, AGFI of 0.90 or greater, CFI of 0.90 or greater, RMSEA ≤ 0.05 and a chi-square that was not significant (*P* > 0.05). Multivariate analyses were conducted using software (SPSS 24.0 J; SPSS Inc. and Amos 24.0 J for Windows, Chicago, IL). In addition, in the multivariable analysis, the total PCB concentration in serum was logarithmically transformed because of skewed distribution.

## Additional Information

**How to cite this article**: Kobayashi, N. *et al*. Factors associated with aberrant imprint methylation and oligozoospermia. *Sci. Rep.* **7**, 42336; doi: 10.1038/srep42336 (2017).

**Publisher's note:** Springer Nature remains neutral with regard to jurisdictional claims in published maps and institutional affiliations.

## Supplementary Material

Supplementary Information

Supplementary Table S3

## Figures and Tables

**Table 1:** Background data of the men with normozoospermia, moderate oligozoospermia and severe oligozoospermia.

	Sperm concentrations			*P*-value
	Normal (n = 151)	Moderate (n = 40)	Severe (n = 30)	
**Sperm parameters**				
Abstinence period (days)	5.1 ± 3.0	5.8 ± 9.2	4.6 ± 2.0	0.506
≤4.0	54.7%	64.1%	43.3%	0.228
4.0<	45.3%	35.9%	56.7%	
Volume (ml)	3.3 ± 1.5	3.3 ± 1.4	3.1 ± 2.0	0.501
≤3.0	50.7%	52.5%	56.7%	0.831
3.0<	49.3%	47.5%	43.3%	
Motility (%)	57.4 ± 15.6	42.9 ± 19.1	26.9 ± 23.1	<0.001**
≤53.0	41.7%	70.0%	90.0%	<0.001**
53.0<	58.3%	30.0%	10.0%	
Malformation (%)	56.5 ± 10.0	60.6 ± 11.1	69.4 ± 11.0	<0.001**
≤58.0	62.7%	47.5%	8.0%	<0.001**
58.0<	37.3%	52.5%	92.0%	
Imprinted methylation (%)				
Normal	83.4%	77.5%	30.0%	<0.001**
Abnormal	16.6%	22.5%	70.0%	
**Physical parameters**				
Age (year)	35.2 ± 5.7	36.4 ± 4.6	36.8 ± 6.5	0.326
≤35	53.0%	42.5%	46.7%	0.456
35<	47.0%	57.5%	53.3%	
Height (cm)	171.0 ± 5.3	171.8 ± 6.2	172.2 ± 6.8	0.642
≤171.0	53.7%	47.5%	50.0%	0.762
171.0<	46.3%	52.5%	50.0%	
Body weight (kg)	70.8 ± 11.3	71.3 ± 10.0	70.6 ± 9.4	0.859
≤70.0	53.7%	45.0%	56.7%	0.550
70.0<	46.3%	55.0%	43.3%	
BMI (kg/m^2^)	24.2 ± 3.6	24.2 ± 2.9	23.8 ± 2.9	0.797
≤23.8	50.3%	45.0%	53.3%	0.765
23.8<	49.7%	55.0%	46.7%	
Sleeping time (hours)	6.7 ± 0.8	6.7 ± 0.8	6.5 ± 0.9	0.408
≤7.0	85.4%	82.9%	88.0%	0.856
7.0<	14.6%	17.1%	12.0%	
Annual income (million yen)	4.8 ± 2.2	5.0 ± 2.3	4.7 ± 1.9	0.921
≤4.0	53.3%	50.0%	52.2%	0.944
4.0<	46.7%	50.0%	47.8%	
Education (%)				
High school or less	30.1%	17.6%	37.5%	0.218
College, University or Graduate school	69.9%	82.4%	62.5%	
Residence (%)				
Farming or fishing village	16.1%	20.0%	8.3%	0.478
Residential district or others	83.9%	80.0%	91.7%	
**Blood parameters**				
LH (mIU/ml)	3.0 ± 1.2	3.3 ± 1.2	5.0 ± 3.8	0.010*
FSH (mIU/ml)	6.7 ± 4.2	8.3 ± 5.3	14.9 ± 12.1	<0.001**
PRL (ng/ml)	9.0 ± 4.2	10.2 ± 3.8	9.1 ± 4.6	0.159
T (ng/dl)	423.7 ± 147.3	446.7 ± 145.3	544.2 ± 263.4	0.136
**Lifestyle parameters**				
Smoking (%)				
Current smoker	22.4%	25.7%	40.0%	0.182
Never smoker or ex-smoker	77.6%	74.3%	60.0%	
Exercise (%)				
Exerciser	48.4%	61.8%	29.2%	0.050
Non-exerciser	51.6%	38.2%	70.8%	

Data are shown as mean ± SD. Numbers within brackets indicate the number of patients studied. All questionnaires were categorized into two groups, and the three classifications were patients with normozoospermia (Normal), moderate oligozoospermia (Moderate) and severe oligozoospermia (Severe). The results of serum pituitary hormone examination are shown as blood parameters (luteinizing hormone (LH), follicle-stimulating hormone (FSH), prolactin (PRL) and testosterone (T)). Significant differences were determined using the chi-squared test or Kruskal-Wallis test. *P < 0.05, **P < 0.01.

**Table 2:** Total PCBs and their isoform concentrations in the three sperm groups.

	Sperm concentrations			*P*-value
	Normal (n = 55)	Moderate (n = 33)	Severe (n = 25)	
**PCB** (**ng**/**g fat**)				
Total PCB	46.8 ± 37.3	74.1 ± 35.0	75.4 ± 50.0	<0.001**
≤51.9	69.1%	30.3%	36.0%	<0.001**
51.9<	30.9%	69.7%	64.0%	
#70/80	3.2 ± 2.6	3.7 ± 1.7	2.9 ± 2.0	0.014*
#99	2.5 ± 1.8	3.0 ± 1.6	3.2 ± 2.4	0.032*
#118	2.8 ± 2.7	3.8 ± 2.2	4.3 ± 3.1	0.001**
#153	8.7 ± 8.6	6.1 ± 5.0	6.3 ± 3.5	0.854
#163/164	11.1 ± 15.2	19.3 ± 10.4	20.3 ± 15.4	<0.001**
#138	6.9 ± 6.5	9.8 ± 5.6	11.3 ± 9.2	0.002**
#182/187	3.0 ± 4.9	10.1 ± 7.0	8.5 ± 7.2	<0.001**
#180	6.7 ± 7.3	13.3 ± 8.0	14.1 ± 9.3	<0.001**
#170	2.1 ± 1.9	5.1 ± 3.1	4.5 ± 3.7	<0.001**

Total serum PCBs and their isoforms were measured in 113 randomly selected subjects by high-resolution gas chromatography and high-resolution mass spectrometry using isotope dilution. Data are shown as mean ± SD. Numbers within brackets indicate the number of patients studied. The three classifications were patients with normozoospermia, moderate oligozoospermia and severe oligozoospermia. Significant differences were determined using the chi-squared test or the Kruskal-Wallis test. *P < 0.05, **P < 0.01.

**Table 3:** Outcome of ART treatment with imprinted methylation and concentrations of sperm.

	**Methylation**						*P*-value
	**Normal (n = 156)**			**Abnormal (n = 63)**			
Pregnancy (%)	32.7%		(51/156)	39.7%		(25/63)	0.325
Miscarriage (%)	10.9%		(17/156)	25.4%		(16/63)	0.007**
Live-birth (%)	21.8%		(34/156)	14.3%		(9/63)	0.205
	**Sperm concentrations**						*P*-value
	**Normal (n = 100)**		**Moderate (n = 79)**		**Severe (n = 40)**		
Pregnancy (%)	40.0%	(40/100)	26.6%	(21/79)	37.5%	(15/40)	0.159
Miscarriage (%)	12.0%	(12/100)	13.9%	(11/79)	25.0%	(10/40)	0.142
Live-birth (%)	28.0%	(28/100)	12.7%	(10/79)	12.5%	(5/40)	0.017*

ART treatments indicates the number of ART treatments. Miscarriages and live births indicate the numbers of miscarriages and live births after ART treatment. The pregnancy rates (pregnancies/ART treatments), miscarriage rates (miscarriages/ART treatments) and live-birth rates (live births/ART treatments) were compared for sperm with and without abnormal imprint methylation (the upper section). In the lower section, the miscarriage rates, live-birth rates and pregnancy rates were compared according to the classification of the sperm. Significant differences were determined using the chi-squared test. *P < 0.05, **P < 0.01.
