# Supplementary material for: Factors associated with aberrant imprint methylation and oligozoospermia
Source: Sci Rep. 2017 Feb 10;7:42336. doi: 10.1038/srep42336 (PMC5301220; doi:10.1038/srep42336)
Supplement: Supplementary Information [file srep42336-s1.pdf]

Supplementary information

Factors associated with aberrant imprint methylation and oligozoospermia

Norio Kobayashi[1,2,#], Naoko Miyauchi[1,#], Nozomi Tatsuta[3], Akane Kitamura[1], Hiroaki Okae[1], Hitoshi Hiura[1], Akiko Sato[1,4], Takafumi Utsunomiya[4], Nobuo Yaegashi[5], Kunihiko Nakai[3], Takahiro Arima[1,*]

[1]Department of Informative Genetics, Tohoku University Graduate School of Medicine, 2-1 Seiryo-cho, Aoba-ku, Sendai, 980-8575, Japan

[2]Laboratory of Animal Reproduction and Development, Graduate School of Agricultural Science, Tohoku University, 1-1 Amamiya-machi, Tsutsumidori, Aoba-ku, Sendai, 981-8555, Japan

[3]Department of Development and Environmental Medicine, Tohoku University Graduate School of Medicine, Sendai, 980-8575, Japan

[4]St. Luke Clinic, Oita, 870-0823, Japan

[5]Departments of Obstetrics and Gynecology, Tohoku University Graduate School of Medicine, Sendai, 980-8574, Japan

[#]These authors contributed equally to this work

[*]To whom correspondence should be addressed
TEL: ++81 22 717 7844;
FAX: ++81 22 717 7063;
Email: tarima@med.tohoku.ac.jp

Supplementary information

1. **Supplemental Figure S1. Aberrant DNA methylation of imprinted loci in sperm from infertile men.** (A) The frequency of abnormally imprinted loci. 24.8% (55/221) were found to have abnormal imprint methylation and about half of the cases with aberrant methylation had it in one locus. (B) The frequency of methylation errors in each imprinted locus. White bars indicate 3 paternally methylated gDMR (pgDMR) and black bars indicate 19 maternally methylated gDMR (mgDMR). The Y-axis indicates the frequency of imprinted methylation errors (%) (C) Association between abnormal methylation samples and the sperm concentration, sperm motility rate and sperm malformation rate. The Y-axis indicates the frequency of patients with abnormal imprint methylation (%) for each sperm parameters. Normal: Normozoospermia (20×106/ml), Moderate: Moderate oligozoospermia (5–20×106/ml), Severe: Severe oligozoospermia (5×106/ml). *P<0.05, **P<0.01.

2. **Supplemental Table S1. Factors associated with the presence and absence of imprint methylation errors.** Data are shown as mean±SD. Numbers within brackets indicate the number of patients studied. *P<0.05, **P<0.01.

3. **Supplemental Table S2. Comparison of the associated factors according to sperm concentration.** Data are shown as mean±SD. Numbers within brackets indicate the number of patients studied. The three classifications were normozoospermia, moderate oligozoospermia and severe oligozoospermia. *P<0.05, **P<0.01.

4. **Supplemental Table S3. Raw data from our study.** Table S3 shows imprinted methylation profiles, sperm properties, lifestyle and food/drink intakes and serum PCBs and hormone levels in each subject. Data are shown as + (≥80% methylation), - (≤20% methylation) and ND (not determined).

Supplemental Figure S1

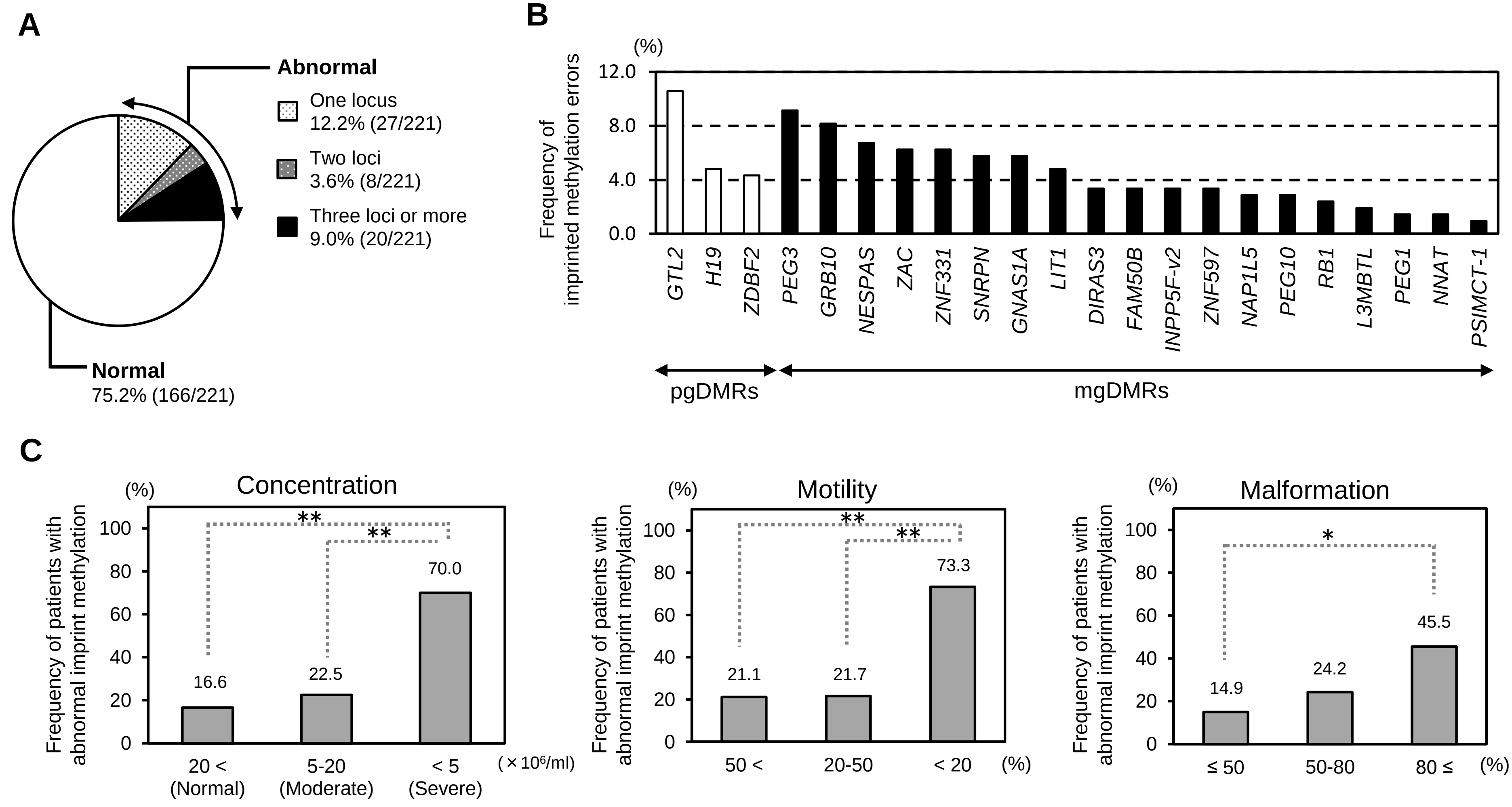

Supplemental Table S1. Factors associated with the presence and absence of imprint methylation errors

	Imprinted methylation		
	Normal (n=166)	Abnormal (n=55)	*P* -value
**Sperm parameters**			
Abstinence period (days)	4.8±2.8	6.0±7.9	0.266
≤4.0	57.3%	47.3%	0.195
4.0<	42.7%	52.7%	
Volume (ml)	3.4±1.5	3.0±1.6	0.094
≤3.0	49.7%	58.2%	0.275
3.0<	50.3%	41.8%	
Concentration (10^6/ml)	50.5±44.5	32.2±41.0	< 0.001 **
<5.0	5.4%	38.2%	< 0.001 **
5.0≤	94.6%	61.8%	
Motility (%)	52.9±19.0	43.9±23.4	0.026 *
≤53.0	49.4%	65.5%	0.039 *
53.0<	50.6%	34.5%	
Malformation (%)	58.4±10.9	60.1±11.8	0.251
≤58.0	55.2%	48.0%	0.375
58.0<	44.8%	52.0%	
**Physical parameters**			
Age (year)	35.4±5.4	36.5±6.1	0.230
≤35	51.8%	45.5%	0.413
35<	48.2%	54.5%	
Height (cm)	171.0±5.8	172.3±5.4	0.223
≤171.0	53.7%	47.3%	0.412
171.0<	46.3%	52.7%	
Body weight (kg)	70.6±11.2	71.6±9.6	0.449
≤70.0	52.4%	52.7%	0.970
70.0<	47.6%	47.3%	
BMI (kg/m^2)	24.1±3.4	24.2±3.4	0.812
≤23.8	50.0%	49.1%	0.907
23.8<	50.0%	50.9%	
Sleeping time (hours)	6.7±0.8	6.6±0.8	0.464
≤7.0	84.2%	88.6%	0.467
7.0<	15.8%	11.4%	
Annual income (million yen)	4.8±2.3	4.7±1.7	0.670
≤4.0	52.6%	52.4%	0.984
4.0<	47.4%	47.6%	
Education (%)			
High school or less	29.2%	27.3%	0.806
College, University or Graduate school	70.8%	72.7%	
Residence (%)			
Farming or fishing village	19.4%	4.5%	0.019 *
Residential district or others	80.6%	95.5%	
**Lifestyle parameters**			
Smoking (%)			
Current smoker	23.6%	31.1%	0.312
Never smoker or ex-smoker	76.4%	68.9%	

Exercise (%)			
Exerciser	53.6%	31.8%	0.012 *
Non-exerciser	46.4%	68.2%	
Materials used in work in the past			
Pesticide (%)			
Used	10.0%	0.0%	0.054
Not used	88.6%	97.7%	
Unknown	1.4%	2.3%	
PCB or dioxin (%)			
Used	4.3%	0.0%	0.527
Not used	87.1%	90.9%	
Unknown	8.6%	9.1%	
Radioactive material (%)			
Used	9.3%	9.1%	0.767
Not used	87.9%	86.4%	
Unknown	2.9%	4.5%	
Things used for more than half a day			
Chlorine bleach, insecticide (%)			
None	87.0%	85.4%	0.791
1-3 times/month or more	13.0%	14.6%	
Medical disinfectant (%)			
None	89.5%	82.9%	0.262
1-3 times/month or more	10.5%	17.1%	
Permanent marker (%)			
None	63.4%	61.0%	0.780
1-3 times/month or more	36.6%	39.0%	
Aqueous magic inks, ink-jet printer (%)			
None	69.9%	65.9%	0.626
1-3 times/month or more	30.1%	34.1%	
Organic solvent (%)			
None	82.9%	85.0%	0.759
1-3 times/month or more	17.1%	15.0%	
Copy machine, laser printer (%)			
None	49.2%	51.2%	0.822
1-3 times/month or more	50.8%	48.8%	
Lubricating oil (%)			
None	91.9%	95.1%	0.496
1-3 times/month or more	8.1%	4.9%	
Insecticide (%)			
None	89.5%	95.1%	0.279
1-3 times/month or more	10.5%	4.9%	
Radioactive material, isotope (%)			
None	95.2%	87.8%	0.102
1-3 times/month or more	4.8%	12.2%	
Dietary habit			
Meals (%)			
Twice per day	23.0%	11.6%	0.105
Three times per day	77.0%	88.4%	
Eat regularly (%)			
Yes	64.0%	69.8%	0.489
No	36.0%	30.2%	

Rice daily averag (%)			
1 bowl/day or less	30.2%	30.2%	0.998
2-3 bowls/day or more	69.8%	69.8%	
Miso soup daily average (%)			
1 bowl/day or less	69.4%	76.2%	0.398
2 bowls/day or more	30.6%	23.8%	
Confections (%)			
1-2 times/week or less	60.7%	61.9%	0.893
3-4 times/week or more	39.3%	38.1%	
Meat (%)			
3-4 times/week or less	74.3%	67.4%	0.382
5-6 times/week or more	25.7%	32.6%	
Fish (%)			
1-2 times/week or less	64.0%	54.8%	0.283
3-4 times/week or more	36.0%	45.2%	
Vegetable (%)			
5-6 times/week or less	52.6%	57.1%	0.606
Every day	47.4%	42.9%	
Fruit (%)			
1-2 times/week or less	73.1%	73.2%	0.996
3-4 times/week or more	26.9%	26.8%	
Fry (%)			
1-2 times/week or less	60.3%	50.0%	0.238
3-4 times/week or more	39.7%	50.0%	
Eat out (%)			
1-2 times/week or less	78.7%	74.4%	0.559
3-4 times/week or more	21.3%	25.6%	
Precooked food (%)			
1-3 times/month or less	57.5%	67.4%	0.245
1-2 times/week or more	42.5%	32.6%	
Soy food (%)			
1-2 times/week or less	48.1%	62.8%	0.094
3-4 times/week or more	51.9%	37.2%	
Vitamin compound (%)			
Currently taking	11.9%	11.6%	0.968
Never take	88.1%	88.4%	
Milk (%)			
1-3 times/month or less	62.7%	61.4%	0.875
1-2 times/week or more	37.3%	38.6%	
Green tea (%)			
3-4 times/week or less	57.0%	45.5%	0.181
5-6 times/week or more	43.0%	54.5%	
Oolong tea (%)			
1-3 times/month or less	57.5%	63.6%	0.470
1-2 times/week or more	42.5%	36.4%	
100% fruit juice (%)			
1-3 times/month or less	76.9%	72.7%	0.578
1-2 times/week or more	23.1%	27.3%	
Vegetable juice (%)			
1-3 times/month or less	78.4%	83.7%	0.447
1-2 times/week or more	21.6%	16.3%	

Lactin acid drink (%)			
1-3 times/month or less	81.3%	75.0%	0.363
1-2 times/week or more	18.7%	25.0%	
Sports drink (%)			
1-3 times/month or less	67.9%	79.5%	0.141
1-2 times/week or more	32.1%	20.5%	
Coffee (%)			
5-6 times/week or less	54.4%	40.9%	0.119
Every day	45.6%	59.1%	
Carbonated drink (%)			
1-2 times/week or less	81.1%	75.0%	0.388
3-4 times/week or more	18.9%	25.0%	
PCB (ng/g fat)			
Total PCB	62.4±41.5	59.7±42.6	0.696
≤51.9	50.0%	50.9%	0.920
51.9<	50.0%	49.1%	
#70/80	3.3±1.9	3.3±2.6	0.399
#99	2.8±2.0	2.7±1.9	0.931
#118	3.2±2.7	3.6±2.7	0.354
#153	7.7±7.3	7.1±6.3	0.476
#163/164	16.7±15.4	14.2±13.6	0.261
#138	8.6±6.6	8.9±7.8	0.968
#182/187	6.5±7.6	6.0±6.1	0.609
#180	10.3±9.1	10.3±8.3	0.745
#170	3.4±3.1	3.6±3.1	0.436

Supplemental Table S2. Comparison of the associated factors according to sperm concentration

	Sperm concentrations			*P*-value
	Normal (n=151)	Moderate (n=40)	Severe (n=30)	
Materials used in work in the past				
Pesticide (%)				
Used	8.0%	8.6%	4.2%	0.950
Not used	89.6%	91.4%	95.8%	
Unknown	2.4%	0.0%	0.0%	
PCB or dioxin (%)				
Used	3.2%	2.9%	4.2%	0.532
Not used	89.6%	88.6%	79.2%	
Unknown	7.2%	8.6%	16.7%	
Radioactive material (%)				
Used	7.2%	17.1%	8.3%	0.175
Not used	90.4%	80.0%	83.3%	
Unknown	2.4%	2.9%	8.3%	
Things used for more than half a day				
Chlorine bleach, insecticide (%)				
None	88.2%	86.7%	79.2%	0.454
1-3 times/month or more	11.8%	13.3%	20.8%	
Medical disinfectant (%)				
None	91.0%	86.7%	75.0%	0.089
1-3 times/month or more	9.0%	13.3%	25.0%	
Permanent marker (%)				
None	66.4%	56.7%	54.2%	0.397
1-3 times/month or more	33.6%	43.3%	45.8%	
Aqueous magic inks, ink-jet printer (%)				
None	74.8%	55.2%	58.3%	0.061
1-3 times/month or more	25.2%	44.8%	41.7%	
Organic solvent (%)				
None	84.7%	83.3%	77.3%	0.657
1-3 times/month or more	15.3%	16.7%	22.7%	
Copy machine, laser printer (%)				
None	53.2%	43.3%	41.7%	0.441
1-3 times/month or more	46.8%	56.7%	58.3%	
Lubricating oil (%)				
None	91.9%	96.7%	91.7%	0.736
1-3 times/month or more	8.1%	3.3%	8.3%	
Insecticide (%)				
None	92.8%	86.7%	87.5%	0.418
1-3 times/month or more	7.2%	13.3%	12.5%	
Radioactive material, isotope (%)				
None	95.5%	86.7%	91.7%	0.168
1-3 times/month or more	4.5%	13.3%	8.3%	
Dietary habit				
Meals (%)				
Twice per day	23.4%	17.6%	8.3%	0.223
Three times per day	76.6%	82.4%	91.7%	
Eat regularly (%)				
Yes	63.7%	73.5%	62.5%	0.538
No	36.3%	26.5%	37.5%	
Rice daily averag (%)				
1 bowl/day or less	32.3%	17.6%	37.5%	0.183
2-3 bowls/day or more	67.7%	82.4%	62.5%	
Miso soup daily average (%)				
1 bowl/day or less	70.2%	76.5%	66.7%	0.698
2 bowls/day or more	29.8%	23.5%	33.3%	
Confections (%)				
1-2 times/week or less	57.5%	66.7%	70.8%	0.361
3-4 times/week or more	42.5%	33.3%	29.2%	

Meat (%)				
3-4 times/week or less	71.9%	70.6%	79.2%	0.734
5-6 times/week or more	28.1%	29.4%	20.8%	
Fish (%)				
1-2 times/week or less	60.8%	70.6%	54.2%	0.416
3-4 times/week or more	39.2%	29.4%	45.8%	
Vegetable (%)				
5-6 times/week or less	52.9%	50.0%	62.5%	0.618
Every day	47.1%	50.0%	37.5%	
Fruit (%)				
1-2 times/week or less	74.6%	64.7%	78.3%	0.436
3-4 times/week or more	25.4%	35.3%	21.7%	
Fry (%)				
1-2 times/week or less	62.8%	50.0%	43.5%	0.133
3-4 times/week or more	37.2%	50.0%	56.5%	
Eat out (%)				
1-2 times/week or less	76.0%	88.2%	70.8%	0.221
3-4 times/week or more	24.0%	11.8%	29.2%	
Precooked food (%)				
1-3 times/month or less	60.0%	60.6%	58.3%	0.984
1-2 times/week or more	40.0%	39.4%	41.7%	
Soy food (%)				
1-2 times/week or less	50.8%	50.0%	58.3%	0.780
3-4 times/week or more	49.2%	50.0%	41.7%	
Vitamin compound (%)				
Currently taking	11.7%	8.8%	16.7%	0.639
Never take	88.3%	91.2%	83.3%	
Milk (%)				
1-3 times/month or less	61.7%	67.6%	58.3%	0.743
1-2 times/week or more	38.3%	32.4%	41.7%	
Green tea (%)				
3-4 times/week or less	55.0%	51.4%	54.2%	0.933
5-6 times/week or more	45.0%	48.6%	45.8%	
Oolong tea (%)				
1-3 times/month or less	59.2%	58.8%	58.3%	0.997
1-2 times/week or more	40.8%	41.2%	41.7%	
100% fruit juice (%)				
1-3 times/month or less	75.8%	73.5%	79.2%	0.885
1-2 times/week or more	24.2%	26.5%	20.8%	
Vegetable juice (%)				
1-3 times/month or less	80.0%	76.5%	82.6%	0.841
1-2 times/week or more	20.0%	23.5%	17.4%	
Lactin acid drink (%)				
1-3 times/month or less	80.8%	85.3%	66.7%	0.194
1-2 times/week or more	19.2%	14.7%	33.3%	
Sports drink (%)				
1-3 times/month or less	72.5%	67.6%	66.7%	0.767
1-2 times/week or more	27.5%	32.4%	33.3%	
Coffee (%)				
5-6 times/week or less	55.4%	48.6%	33.3%	0.135
Every day	44.6%	51.4%	66.7%	
Carbonated drink (%)				
1-2 times/week or less	81.5%	84.8%	62.5%	0.077
3-4 times/week or more	18.5%	15.2%	37.5%	
